# Functional Diversity of Serotonin Neurons in the Dorsal and Median Raphe Nuclei in Emotional Responses

**DOI:** 10.1002/npr2.70015

**Published:** 2025-04-20

**Authors:** Yu Ohmura, Kazuki Nagayasu

**Affiliations:** ^1^ Chinese Institute for Brain Research, Beijing (CIBR) Beijing China; ^2^ Laboratory of Molecular Neuropharmacology Graduate School of Pharmaceutical Sciences, Osaka University Suita Japan; ^3^ Project for Neural Networks Graduate School of Pharmaceutical Sciences, Osaka University Suita Japan

**Keywords:** 5‐HT, antidepressants, midbrain raphe

## Abstract

Of serotonergic nuclei in the central nervous system, mainly the dorsal raphe nucleus (DRN) and median raphe nucleus (MRN) project to the forebrain and midbrain; therefore, these nuclei are involved in emotional/cognitive functions and psychiatric disorders. Researchers have often generalized findings from the DRN to represent the functions of the entire serotonergic system, primarily due to the fact that the DRN is the largest serotonergic nucleus and due to the assumption that the serotonergic system operates as a single, cohesive unit. However, recent evidence is challenging this perspective and necessitating a reevaluation. In this brief review, we summarize recent studies demonstrating the functional diversity of the DRN alongside the functional unity of the MRN. These findings suggest that different subpopulations within the serotonergic system may exert opposing effects on emotional functions. Furthermore, this diversity‐aware approach will help settle ongoing debates regarding the serotonin hypothesis of depression, which stems from the difficulty in the application of this approach in humans. We advocate for increased efforts to identify factors associated with these functional subgroups, which could lead to more targeted and effective interventions.

## Abstract 2

Recent advances challenge the traditional view of serotonin as a uniform neuromodulatory system, revealing the functional diversity of serotonin neurons in raphe nuclei, including the dorsal (DRN) and median raphe nuclei (MRN). This review highlights how distinct serotonergic subpopulations exert opposing effects on emotional regulation, necessitating a reevaluation of the serotonin hypothesis of depression. By identifying functionally distinct serotonergic subgroups, researchers can develop more targeted interventions, moving beyond nonselective serotonin modulation toward precision treatments for psychiatric disorders.

## Graphical abstract

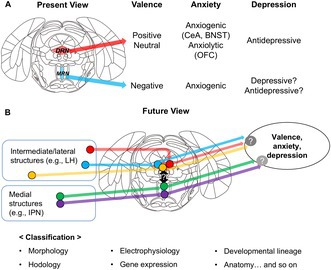

## 1 Introduction

Drugs that modulate serotonergic neurotransmission including selective serotonin reuptake inhibitors (SSRIs), 5‐HT_1A_ partial agonists, and 5‐HT_2A_ antagonists, have long occupied a central role in the treatment of psychiatric disorders. However, serotonin studies involving humans have not always been consistent, and whether the central serotonergic system remains a promising therapeutic target is debated [1, 2]. In contrast, because of remarkable technological developments in recent years, preclinical evidence has accumulated and relatively converged.

**FIGURE 1:**
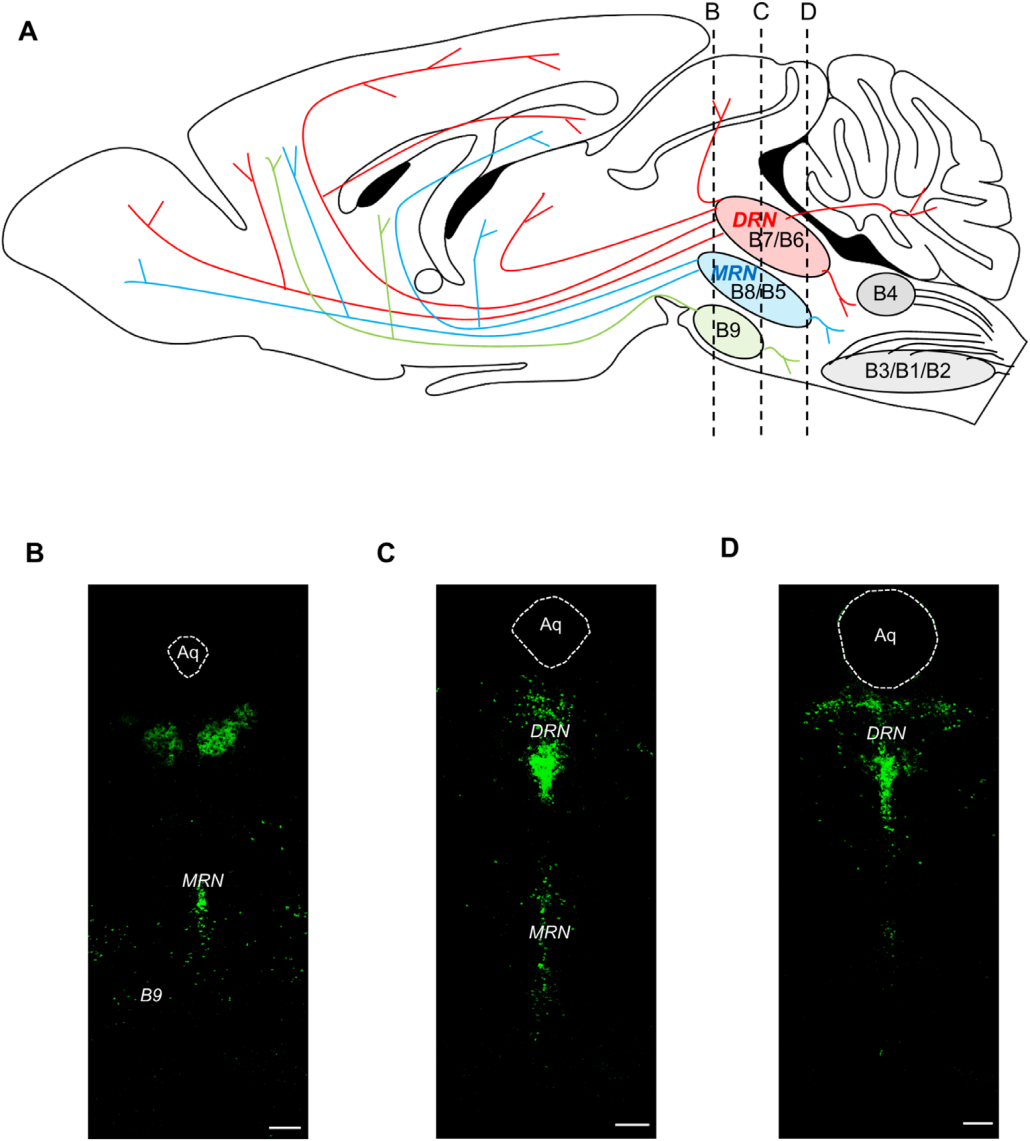
Sagittal and coronal views of the central serotonergic system in mice. (A) Schematic drawing of the sagittal view of the central serotonergic system. We described the patterns of serotonergic projections based on Jacobs and Azmitia [3] and Muzerelle et al. [4]. (B–D) Representative examples of coronal sections containing serotonin neurons. Green colors indicate TPH2 (a marker of serotonin neurons) staining. The immunohistochemistry procedure was described in Kawai et al. [5]. Aq, Aqueduct. White bar = 200 μm.

The central serotonergic system comprises nine nuclei (B1–B9) (Figure 1), of which the dorsal raphe nucleus (DRN, B6 and B7), median raphe nucleus (MRN, B5 and B8), and supralemniscal nucleus (B9) project to the forebrain and midbrain in rodents [4], similar to humans [6]. Consistent with this anatomical connection, the DRN and MRN are involved in emotional and cognitive functions [7, 8].

Researchers have implicitly assumed that examining the DRN allows us to understand most functions of serotonergic systems because the DRN is the largest nucleus among the serotonergic origins [3]. Some people have considered that rostrally projecting serotonergic systems are functionally consistent regardless of the location, and even if there are slight functional differences between locations, their functions will not contradict each other. Indeed, the number of publications focusing on serotonin neurons is clearly biased to those in the DRN. We found 3310, 571, and 7 publications when we searched PubMed using the terms “dorsal raphe nucleus,” “median raphe nucleus,” and “supralemniscal nucleus,” respectively, on September 8, 2024. Although we have not investigated other names for these nuclei, they are the most common. Moreover, researchers have often concluded the role of the serotonergic system in a particular function even when they only targeted the DRN.

However, recent findings using optogenetics, chemogenetics, and imaging have urged us to revise this view in two ways: (1) the serotonergic system projecting to the forebrain and midbrain is more diverse than previously thought and even has opposing functions in different subpopulations and (2) researchers need to pay more attention to the MRN to understand the serotonergic system from the perspective of searching for therapeutic targets.

## 2 Functional Diversity of Serotonin Neurons in the DRN

Some astute researchers have highlighted the possibility that the function of the DRN is not uniform even for closely related emotions for over 30 years: the activation of serotonin neurons in the DRN could enhance anxiety while attenuating panic [9]. Subsequent research supported most parts of the Deakin/Graeff hypothesis [8]. They called it the “dual” role of the DRN serotonergic system. However, recent studies suggest they are more than dual.

Recent studies using optogenetics and chemogenetics have demonstrated that the activation of serotonin neurons in the DRN, particularly those projecting to the ventral tegmental area, exerts antidepressant‐like and rewarding effects [10, 11, 12, 13, 14, 15, 16].

Unit recording and Ca^2+^ imaging studies also suggest the existence of subpopulations with distinct patterns of serotonin neural activity in the DRN: they respond differentially to reward and aversive stimuli [17, 18, 19].

Thus, the idea that the serotonergic system is a single unit is too far from reality, as Deakin and Graeff had already indicated. Furthermore, the results of recent studies do not contradict the Deakin/Graeff hypothesis but rather require us to expand their idea more. Even when limited to emotional functions, the functions of serotonin neurons in the DRN are highly diverse, with subpopulations having opposing functions.

## 3 Convergent Evidence Regarding the MRN Function

The role of the MRN serotonin neurons in emotion is somewhat more unified than that of the DRN. Classic lesion and pharmacological studies demonstrated that the inactivation of MRN exerted anxiolytic effects [8]. Consistent with them, recent studies have shown that optogenetic activation of serotonin neurons in the MRN induces anxiety‐like behavior, avoidance behavior, and reduction in reward responses [5, 15, 20, 21]. Accumulated evidence consistently suggests that the serotonergic system in the MRN plays a facilitatory role in aversive responses. Although further studies demonstrating the roles of MRN unveiled in rodents match those in humans are necessary, it is unreasonable to conclude the role of the central serotonergic system in a particular emotional function by targeting the DRN only, and we have to pay more attention to the functions of MRN.

## 4 Implications for the Serotonin Hypothesis of Depression and Future Directions

These revised views could provide insight into the serotonin hypothesis of depression. As the central serotonergic system has diverse roles in emotions and even opposing functions in different subpopulations, the hypothesis that nonselective reduction of serotonin levels in the whole brain could cause depression no longer makes sense. This is because increasing or decreasing the overall serotonergic activity will cause the different subpopulations to interfere with each other, resulting in inconsistent findings and interpretations [1, 2]. Whether this hypothesis is supported or rejected, the diversity of the serotonergic nervous system indicates that definitive results cannot be obtained if nonselective interventions are used.

Furthermore, arguing based on a hypothesis that cannot be clearly resolved could be unproductive. Assume that the hypothesis is supported with a small effect size. Such a small effect size will not justify the development of agents nonselectively acting on the entire serotonergic system. Meanwhile, assume that the hypothesis is barely rejected. The rejection does not deny the importance of the central serotonergic system in emotional functions, as manipulation of a specific subpopulation in the serotonergic system is already known to significantly affect emotional responses. Whether this hypothesis is correct or not, we need to look for ways to selectively manipulate the subpopulations to more effectively treat psychiatric disorders. If the future direction is the same, whether the hypothesis is right or wrong, then debating it further would not be useful.

**FIGURE 2:**
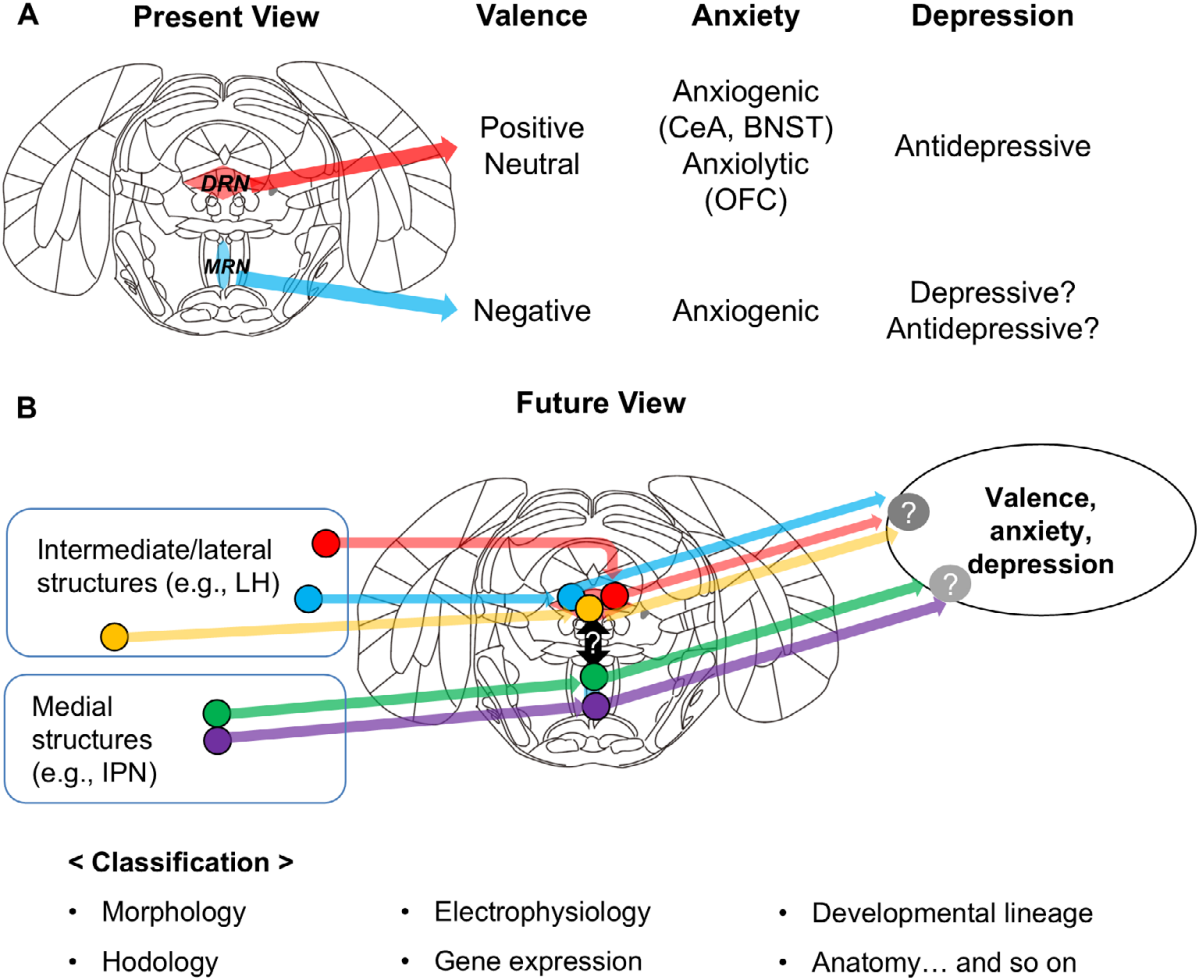
Present and future views of functional roles and classifications of central serotonergic systems. (A) Present view indicates that the functions of the DRN subpopulations and MRN are sometimes opposite to each other. However, it remains unknown whether the DRN and MRN directly regulate each other's activities. CeA: Central nucleus of the amygdala, BNST: Bed nucleus of stria terminalis, OFC: Orbitofrontal cortex. (B) In future studies, we need to consider several other viewpoints to classify the subpopulations. We described the patterns of input based on Ogawa et al. [22] and Pollak Dorocic et al. [23]. However, it does not mean these inputs are mutually exclusive. LH: Lateral hypothalamus, IPN: Interpeduncular nucleus. Modified schematic is from the mouse brain atlas [28].

We suggest ending this fruitless debate and moving toward a more productive direction. Our efforts should focus on finding factors corresponding to functional subgroups and ways to selectively manipulate them. Even a simple distinction (DRN vs. MRN) might be useful, considering that the DRN and MRN have moderately different input sources [22, 23] and projection destinations [4]. Generally, the effect size would decrease with the increase in treatment selectivity. However, given the opposite roles of the DRN and MRN, the effect size on selectivity may follow an inverted‐U shape. We suggest human studies to begin with this simple distinction, similar to others [24]. However, it is far from the exact functional classification of serotonin neurons. Okaty et al. [25] suggested that serotonin neurons can be classified from the viewpoints of morphology, hodology, electrophysiology, gene expression, developmental lineage, and anatomy, including DRN and MRN subregions. It is unclear to what extent each classification corresponds to a functional subpopulation, but any classification is unlikely to perfectly correspond to a function. Therefore, some researchers are attempting to combine two or more of these classifications to achieve functional classification [26, 27]. We believe that preclinical studies should follow these attempts (Figure 2).

## 5 Implications From the MRN Function

The aforementioned animal studies may also explain why SSRIs exert insufficient therapeutic effects on some patients with major depressive disorder but are effective for the others. Our study showed that activation of serotonin neurons in the MRN suppressed the pleasure of taking palatable reward [5], consistent with the fact that SSRIs are not as effective against anhedonia, a symptom of depression [29]. Moreover, the side effect of increasing anxiety often occurs during the initial phase of SSRI administration [30], consistent with the results of mouse studies reporting that MRN activation exerted anxiogenic‐like effects [15, 21]. Thus, the effects of SSRIs on the brain regions targeted by serotonergic terminals originating from the MRN could decrease the antidepressant effects of SSRIs.

A positron emission tomography study reported that relative SERT availability in the terminal region to that in the MRN (somatodendritic) was positively correlated with SSRI treatment response [31]. Somatodendritic SERT mitigates feedback inhibition of serotonin neurons, resulting in a higher serotonergic activity. High MRN serotonin neuron activity may decrease the efficacy of SSRIs, which indicates the potential of selective inhibition of MRN serotonin neurons to overcome treatment‐resistant depression. To achieve this, we propose determining druggable genes, such as receptors, channels, and enzymes, showing biased expression among DRN and MRN serotonin neurons and their subpopulations via single‐cell analysis and immunoprecipitation of mRNA in a cell‐type‐specific manner in stressed animals and healthy controls [25, 32].

## 6 Limitations

The possible reason why the functions of the MRN appear to be unified is that it has been less investigated than the DRN. In fact, some researchers have suggested the existence of subgroups of MRN serotonin neurons [25, 33]. Further studies are warranted to confirm whether they are functionally differentiated.

As aforementioned, many studies have reported that acute MRN activation induces aversive responses. However, it is noteworthy that some researchers suggest that long‐term activation of the MRN may promote stress resilience [9]. Although this theory has insufficient evidence, it deserves to be reexamined using recent sophisticated methods.

This review mainly discussed the functions of serotonin neurons in the DRN and MRN, but it should be noted that there are various cell groups other than serotonin neurons in these nuclei (e.g., glutamate) [34].

This review did not discuss the functions of B9 owing to the lack of information on this brain region. Nevertheless, this region is worth examining.

Finally, although many of the aforementioned studies employed artificial stimulation to examine the roles of serotonin neurons, this technique may not entirely reflect the physiological roles of the manipulated neurons, as demonstrated in recent studies [35]; therefore, the results need to be interpreted with caution.

## 7 Conclusions

Our main messages in this review are as follows:

1. The functions of the serotonergic system are diverse and even opposing in different subpopulations.
2. Therefore, we suggest stopping the debate on the serotonin hypothesis of depression and instead making more efforts to seek factors, especially druggable ones, corresponding to functional subgroups and thereby developing ways to manipulate them selectively.
3. We suggest examining the MRN more from the perspective of developing therapeutics because accumulated evidence consistently suggests the facilitatory role of MRN in aversive responses.

## Author Contributions

**Yu Ohmura:** writing – original draft [lead], writing – review and editing [equal]. **Kazuki Nagayasu:** writing – original draft [supporting], writing – review and editing [equal].

## Conflicts of Interest

The authors declare no conflicts of interest.

## Supporting information


Figure S1.

Figure S2.

Figure S3.

Figure S4.

## Data Availability

Figure source files are provided as [Link], [Link], [Link], [Link].
