# Supplementary material for: Functional Diversity of Serotonin Neurons in the Dorsal and Median Raphe Nuclei in Emotional Responses
Source: Neuropsychopharmacol Rep. 2025 Apr 20;45(2):e70015. doi: 10.1002/npr2.70015 (PMC12010045; doi:10.1002/npr2.70015)
Supplement: Supplementary file 1 — Figure S1. [file NPR2-45-e70015-s002.pptx]

## Slide 1
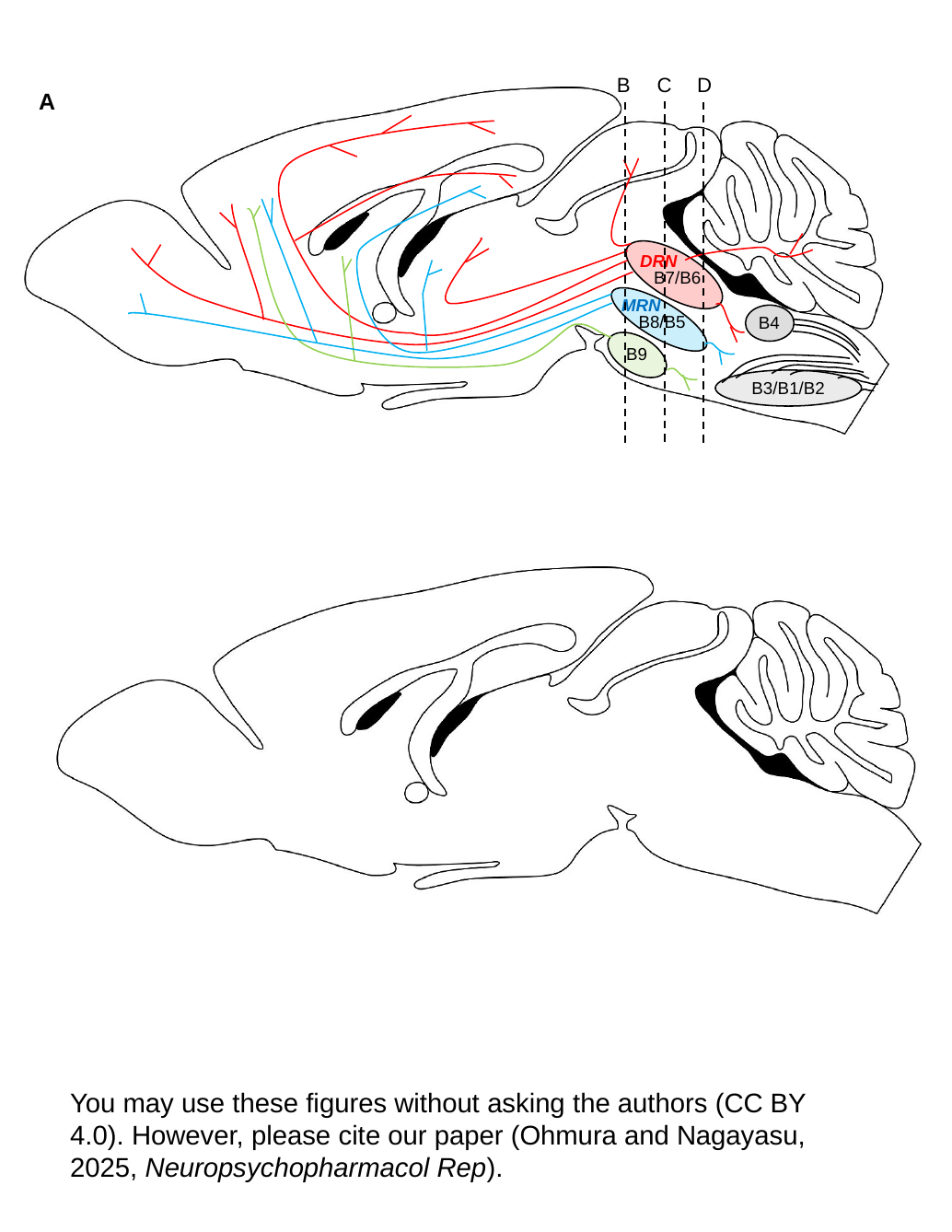

C
B
D
DRN
B7/B6
MRN
B8/B5
B4
B9
B3/B1/B2
A
You may use these figures without asking the authors (CC BY 4.0). However, please cite our paper (Ohmura and Nagayasu, 2025, Neuropsychopharmacol Rep).
