# Supplementary figures and images for: Functional Diversity of Serotonin Neurons in the Dorsal and Median Raphe Nuclei in Emotional Responses
Source: Neuropsychopharmacol Rep. 2025 Apr 20;45(2):e70015. doi: 10.1002/npr2.70015 (PMC12010045; doi:10.1002/npr2.70015)

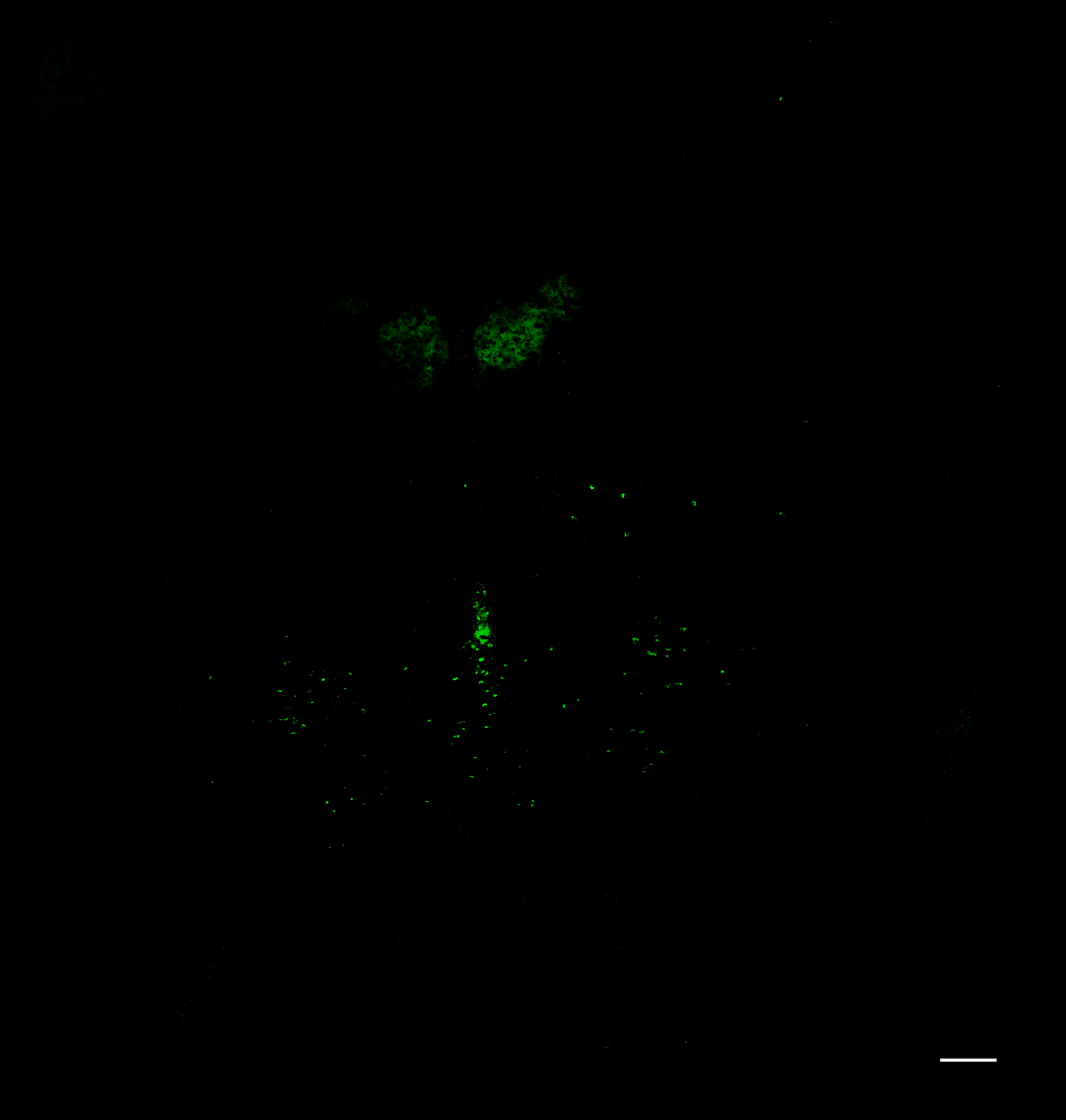

Supplement: Supplementary file 2 — Figure S2. [file NPR2-45-e70015-s001.tif]

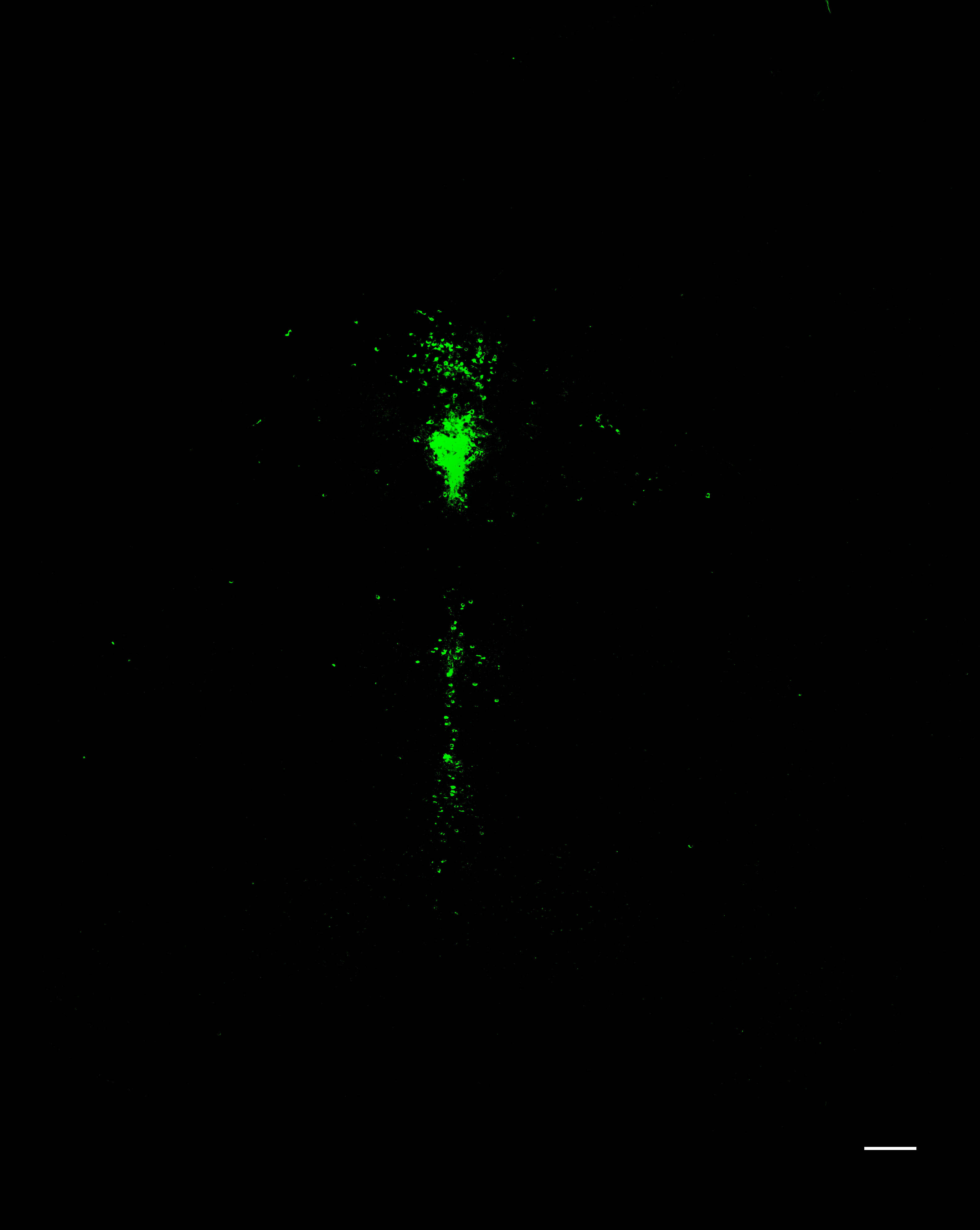

Supplement: Supplementary file 3 — Figure S3. [file NPR2-45-e70015-s004.tif]

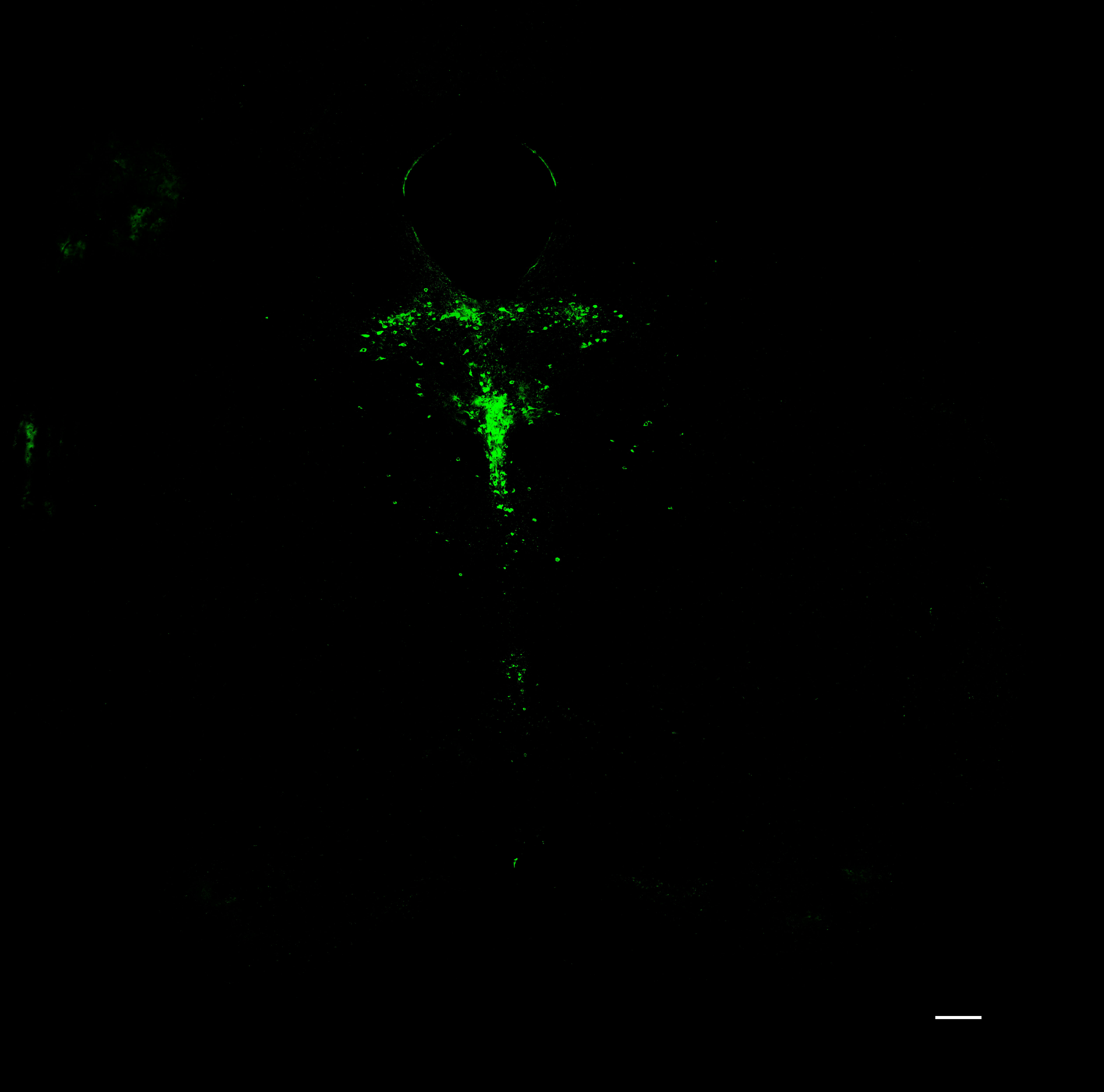

Supplement: Supplementary file 4 — Figure S4. [file NPR2-45-e70015-s003.tif]
